# Book Reviews

**Published:** 1913-03

**Authors:** 


### BOOK REVIEWS

A New Work on the History of Medicine.—W. B. Saunders Company, publishers, of Philadelphia and London, have in active preparation a work on the History of Medicine by Dr. Fielding H. Garrison, for twenty years Principal Assistant Librarian, Surgeon-General's Office, and Editor of the Index Medicus.

His book will present the history of medicine from the earliest ancient and primitive times; on through Egyptian, Sumerian, Oriental, Greek, Byzantine, Mohammedan and Jewish Periods, the Mediaeval Period, the Renaissance, into the Twentieth Century. There will be Appendices covering Medical Chronology, Histories of Important Diseases, Histories of Drugs and Therapeutic Procedures, Histories of Important Surgical Operations and Bibliographic Notes for Collateral Reading.

The illustrations are intended to stimulate the reader's interest in the picturesque aspects of medicine and in the personalities of its great leaders. The biographies will be confined to the most important facts and to interesting personal traits. The original bibliographic references to the important discoveries, operations and experiments will be given. Each period is to be followed by a brief survey of its social and cultural phases.

---

A Reference Handbook of the Medical Sciences, embracing the entire range of Scientific and Practical Medicine and Allied Science. By various writers. Third edition, completely revised and rewritten. Edited by Thomas Lathrop Stedman, A. M., M. D. Complete in eight volumes. Volume one, illustrated by numerous chromolithographs and 611 fine half-tone and wood engravings; 936 pages; imperial quarto. The set, muslin, $56.00; leather, $64.00; half morocco, $72.00 (subscription). Wm. Wood & Co., Publishers, New York.

The first edition of this work was the beginning of our medical library, after the usual college text books. While it has always been our policy to sell, exchange or give away medical books after three or four years, the Reference Hand Book has been retained. Merely for the anatomy and historic matter contained, it is still useful. In a quarter of a century, not only has it been necessary to prepare a work which retains only the frame of the original edition but the labor of architect and builders has passed to other hands. Only occasionally does the present list of contributors contain the names of those who made the first edition. In our own territory, we note the names of M. D. Mann and A. L. Benedict of Buffalo; also those of Julius Pohlman, lately deceased, and of M. A. Crockett, formerly of Buffalo, now of Bedford City, Va. While necessarily expensive, this work is economic, on account of its thoroughness and broad scope.

The Practice of Urology.. A Surgical Treatise on Genitourinary diseases, including Syphilis, by Charles H. Chetwood, M. D., LL. D., Professor of Genitourinary Surgery, New York Polyclinic; Visiting Genitourinary Surgeon to Bellevue Hospital, etc., etc. One volume of 824 pages, large octavo, profusely illustrated by line and half-tone cuts, and by six full-page colored plates. Muslin, $5.00 net; half morocco, $6.00 net. Wm. Wood & Co., Publishers, New York.

No attempt has been made to give an appearance of completeness by mere bulk of matter. On the contrary, the author has condensed as much as possible without omitting important details. Very wisely, he has avoided prolix discussion of moot points, and he has not construed the word *original* to mean an undue presentation of personal views but has drawn freely and properly upon the general fund of knowledge. The publishers are to be congratulated also on the high grade of mechanic work everywhere manifest.

---

Laboratory Data From the Department of Experimental Medicine.. Parke, Davis & Co., Detroit. (Phylacogens).

This is an elaborate report, which will, doubtless, be sent to any physician on request and which is well worth the careful attention of the profession.

---

Golden Rules of Surgery, by the late Augustus Charles Bernays, A. M., M. D., F. R. C. S., second edition, revised by Wm. Thomas Coughlin, M. D., of St. Louis. Published by the C. V. Mosby Co., St. Louis; 280 pages, $2.25.

The exhaustion of the entire first edition speaks well for the popularity of the original author. It is no disrespect to his memory to say that the omission from this edition of some general dissertations valuable as contributions to journals but scarcely appropriate in a text book, as well as the insertion of items inevitably overlooked and the addition of new chapters, has greatly improved the work.

---

Handbook of Diseases of the Rectum, by Louis J. Hirschman, M. D., President of the American Proctologic Society, Lecturer on Rectal Surgery and Clinical Professor of Proctology, Detroit College of Medicine. Revised and rewritten second edition, 338 pages; royal octavo; 172 illustrations, including four colored plates. Price $4.00.

This author also has had the practical compliment of an exhausted first edition. While scholarly and thorough, the book is written especially in the interests of the general practitioner—or, rather, in the interests of the patients who, from the indifference to rectal diseases often displayed by the regular physician, have drifted into the hands of quacks, sometimes unscrupulous, sometimes incompetent, sometimes both. The discussion of office treatment, including local anaesthesia, is especially valuable.

Practical Medicine Series, Vol. 10 of 1912 series. Nervous and Mental Diseases. Hugh T. Patrick and Peter Bassoe of Chicago. 236 pages, illustrated. $1.2[illegible]. ($10 for the entire series of 10 volumes.) The Year Book Publishers, Chicago.

This review of periodic literature is now familiar to all, each series running through approximately a year and appearing in different colored binding to facilitate reference. The thoroughness of the reviews makes the publication valuable for more than temporary reference. A little personal experience well illustrates this fact. Several years ago, in tracing the literature of a subject, we turned down pages for reference. After a while we realized that so many pages were dog-eared as seriously to mar the appearance of the book and to interfere with its closing. But that book is still practically used for the references. The editor of this volume has so thoroughly reviewed the literature on poliomyelitis that we can pardon him for giving his own title as Professor at the Chicago Policlinic.

---

Manual of Elementary Zoology. L. A. Borradaile, M. A., Cambridge. Published by the Oxford University Press. (American Branch, 35 West 32d street, New York.) 470 pages, 301 illustrations. $3.75.

After a general discussion of the animal organism, the author develops the subject by careful analysis of type animals, beginning with the frog. Zoologic classification and evolution is thus left for presentation toward the close of the work, when the student has been prepared for it and can better appreciate the system of naturalists. From a cursory view, many would doubtless regard the book as unsystematic, whereas, the very fact that a natural rather than an artificial method is employed in developing the student's powers of observation, skill in dissection and in laying a foundation of facts for theory, indicates the keenest analysis and highest form of pedagogy.

---

The Surgical Clinics of John B. Murphy, M. D., at Mercy Hospital, Chicago. Vol. 1. No. 6. December, 1912. Published bi-monthly by the W. B. Saunders Co. $8.00 per year.

This issue is devoted mainly to conditions of the bones, joints and cartilages, but covers a variety of other subjects, as mammary cancer, salpingitis and a contribution on the use of radioactive substances in malignant conditions by Dr. Albert Caan. Dr. Murphy's safety razor scalpel is included among the illustrations.

MINOR SURGERY, by Leonard A. Bidwell, F. R. C. S., London. Published by the University of London Press. (Oxford University Press, American Branch, 35 West 32d street, New York.) 299 pages, 129 illustrations. $3.75.

The dividing line between major and minor surgery is somewhat arbitrary, and the author interprets his subject sufficiently broadly. Antiseptics, the preparation of sutures, ligatures, splints, plaster and glass bandages, etc., are included. The various minor surgical conditions are well treated. The prescription of trusses, asphyxia, bed sores, blisters, vaccination, hypodermic injections, including 606, aspiratons and the obtaining of blood for the Widal, Wassermann and opsonic tests, tuberculin vaccinations, etc., render the work especially valuable for the practitioner who does not aspire to being a surgeon.

---

THE MEDICAL DISEASES OF CHILDREN, by T. Rowland C. Whipham, M. A., M. D., M. R. C. P., London. Published as above 417 pages, 67 illustrations. $3.75.

The usual tables of dentition, weight, height, etc.; methods of feeding, etc., are given very briefly. Various diseases are discussed systematically.

---

DISEASES OF THE SKIN, by Willmott Evans, M. D., B. S., B. Sc., F. R. C. S., London. Published as above. 375 pages, 32 illustrations. $3.75.

The first seven chapters are devoted to a general consideration of the subjects, with classification ,methods of diagnosis, etc. Individual diseases are then treated in order.

---

A COMPEND OF HISTOLOGY: Henry Erdmann Radasch, M. S., M. D., Philadelphia, P. Blakiston's Son & Co., Philadelphia; 363 pages, illustrated, $1.25.

This is one of the familiar brown-covered series of Quiz Compends, being the third edition of the volume on Histology. Many a physician would have failed to get his diploma and license; many a man occupying a government position would be in private practice if it were not for such books as these—and this is the practical answer to the critics who object to such aids to learning, on the ground of superficiality and preparation for examination rather than the demands of science and humanity. Like the rest of the series, the book is well printed and displays careful attention to detail, discrimination in omitting non-essentials, and systematic arrangement, on the part of the author.

The Evil Eye, Thantology and Other Essays. Roswell Park, M. D., LL. D. (Yale) of Buffalo. Published by Richard G. Badger, The Gorham Press, Boston; 380 pages, $1.50.

This collection of avowedly non-technical essays is dedicated to Sir William Osler, most appropriately, since the latter has also shown a great interest in medical history and various phases of scholarly research suggested by practical professional experiences and studies. The list of chapters, including Serpent Myths, Iatro-Theurgic Symbolism, The Relation of the Grecian Mysteries to the Foundation of Christianity, . . . . . The Story of the Discovery of the Circulation and others, suggests an otium cum dignitate well spent. But, from personal acquaintance, we know that Dr. Park has not reached this stage, and we should be surprised if he ever does. Moreover, as one follows the stories, there is a wealth of suggestion and an illumination from various angles of practical experience that prompts an apology for the use of the word *scholarly*, so much has this word been misused. These essays do not represent the use of leisure to prevent ennui, but the projection of the learning and powers of observation of an extremely busy and practical man beyond the routine limitation of his tasks into regions of philosophy which greatly need the addition of a little pragmatism. If all essays on philosophic and historic subjects would be written by men equally well grounded in practical knowledge and with minds matured by hard work, the true value of these subjects would be better appreciated.

---

Skin Grafting: Leonard Freeman, M. A., M. D., Denver. C. V. Mosby Co., St. Louis; 139 pages, 24 illustrations, $1.50.

This is a practical monograph, systematic and up-to-date. It may not be out of place, as affording a measure of value to suggest that it is a matter of business for any physician, whether a surgeon or not, to buy this book if he has or expects to have a patient in whose case the question of skin grafting is even sub judice.

---

Complete Catalogue of Medical Publications, The Rebman Co., 1123 Broadway, N. Y.

E. Merck's Annual Report of Recent Advances in Pharmaceutic Chemistry and Therapeutics, Vol. 25, Merck & Co., 45 Park Place. N. Y.

These two commercial publications are free to the profession, except that 15 cents should be sent for postage on the latter. They are valuable for reference.

Atlas of Differential Diagnosis of Diseases of the Nervous System, Dr. Henry Hun of Albany. Published by the Southworth Co. of Troy; 290 pages, illustrated, $4.00.

This work consists almost entirely of large charts, tabulating nervous diseases from various standpoints. The first nine charts work from histories, signs, and symptoms, including those developed by electric tests and examinations of cerebro-spinal fluid, to diagnoses. The remaining thirteen charts analyse disease according to prominent diagnostic points, as motor paralysis, convulsion and spasm, disorders of speech and gait, etc. The tabular views suggest a cross between a system of qualitative chemic analysis and a genealogic chart. In our own early days, we spent hours, arranging tables of almost every conceivable medical topic, but we must confess to a growing dislike for "system" and a preference for familiarization with medical science unit by unit, without trying too hard to bring the various units into relation with one another. Still, there is a great deal to be said in favor of tabular reviews and Dr. Hun's work is elaborate and thorough. A word of warning may be in order. Such charts should not be followed blindly, like a series of guide posts along branching and forking roads. The student should preserve his sense of direction and should grasp the wonderfully complete analytic system by which the author has mapped out the geography of nervous diseases.

---

Treatment After Operation: William Turner, M. S., F. R. C. S., and E. Rock Carling, B. S., F. R. C. S., with a chapter on the eye by L. V. Cargill, F. R. C. S., all of London. University of London Press; Oxford University Press, American Branch, 35 W. 32, N. Y.; 247 pages, illustrated, $3.75.

After discussing anaesthesia, sepsis, haemorrhage, etc., various classes of operations are grouped according to systems and organs and the various surgical risks, methods of prophlaxis, and special points in dressing, nursing, use of postures, etc., are considered. The growing literature on the after-care of operative cases, is a valuable addition to the library of the surgeon and the general practitioner.

---

The Practical Medicine Series; Volume IX; Skin and Venereal Diseases, edited by Dr. William L. Baum of Chicago; Miscellaneous Topics (History, Education, Insurance, Eugenics, etc.), Dr. Harold N. Moyer, Chicago; 237 pages, illustrated, $1.25. Price for the series of 10 volumes, $10.00.

This volume is the next to the last of the 1912 series. As a review of current literature, the Year Book Publishers present the most practical and scientific now available.

TREATMENT OF DISEASE IN CHILDREN, by G. A. Sutherland, M. D., F. R. C. P., London. Published as above. 403 pages, unillustrated. $3.75.

We venture to make a general suggestion that the heading of pages with the general topic of the chapter would facilitate reference. The arrangement of the work requires no comment, but the title is significant as indicating that the author realizes that he is not dealing with a distinct branch of medicine but with ordinary conditions requiring some modification of methods of diagnosis and treatment because of the peculiarities of the immature human being. The great value of the book consists in this realization and in the practical experience of the author.

---

CANCER, THE PROBLEM OF ITS GENESIS AND TREATMENT, by F. W. Forbes Ross, M. D., F. R. C. S., D. P. H., London. Published by the Methuen Co., Ltd., 36 Essex street, W. C., London. 260 pages. $1.25.

Without attempting to review in detail the original and still debatable theories of the author, it may be said that he lays great stress on the relative amounts of various chemicals, as calcium, potassium, iodin, etc., in different bodies, and the metabolic activities upon which such differences depend. Considerable stress is laid on apparent and sometimes unintentional cures of cancer by potassium inrichment, and the author describes various cases purposely treated with potassium and radium emanations. He explains the greater liability of women to cancer as due to physiological functions tending to drain potassium from the system. The apparent relative immunity to cancer conferred by cannibalism, in man and the lower animals, is criticised by allusion to exceptions to the law laid down by some and is explained as due to metabolic and dietetic factors.

---

SOME DON'TS, MEDICAL AND SURGICAL. Pamphlet. Published by the Fellows Co. of New York City.

These Dont's are compiled from authentic sources, as shown by the bibliography of articles consulted, which include the one on Neosalvarsan, by Major Quinton, in our issue of August, 1912. Appendicitis, cancer, cardiac conditions, the stomach, genito-urinary diseases and syphilis are included. This pamphlet will be sent to members of the profession and is worthy of careful reading. It concludes with a good many Do's from eminent foreigners, regarding their advice as to Fellows' Syrup of Hypophosphites.

Diet and Hygiene in Diseases of the Skin, by L. Duncan Bulkley, A. M., M. D., New York. Published by Paul B. Hoeber, 69 East 59th street, New York. 194 pages. $2.00.

This consists of a series of lectures in which the reciprocal relations of articles of food and skin diseases, the effect of various hygienic measures, including the use of waters, courses at springs and baths, are discussed in detail. It is a most valuable work, but unfortunately, difficult to analyse briefly. We commend it to both specialist and general practitioner.

---

Differential Diagnosis, Diseases of the Nervous System. Pamphlet published by the Arlington Chemical Co. of Yonkers, N. Y.

Tabular reviews of symptoms and signs are illustrated by four elaborate colored plates, including 17 views of the brain and cord in diseased states. This is a valuable epitome of diagnosis, issued with the compliments of the company.

---

Diagnostic Points in Gastro-Intestinal Disease. Pamphlet published by the New York Pharmacal Association, Yonkers, N. Y., for complimentary distribution.

This is a book of 45 large pages, copiously illustrated. The nine colored pictures of oesophagoscopic views, the page of new instruments, for examination of the oesophagus, duodenum and stomach, the microscopic features of stomach contents and faces, and the illustrations of intestinal abnormalities are particularly noteworthy. The text very concisely states a wide range of diagnostic points. Altogether, this work is one of the most ambitious and valuable of its kind.

---

Blood Pressure, Technique Simplified, by Dr W. H. Cowing, Rochester. Published by Tycos Instrument Co., Rochester. 122 pages, illustrated.

This is one of the best of the publications of its kind, serving the legitimate purpose of facilitating the use of the instruments of the Taylor Instrument Companies, and, at the same time, presenting in convenient form the theory, observed facts, and technique of blood pressure examinations. The word *Tycos* is Greek, means a mason's pick or hammer and alludes to the stroke of the pulse. This etymologic explanation requires only a brief note of qualification, to the effect that the word was manufactured by running together part of the letters of the name of the firm, some years before we studied out the classic etymology.

